# *P. acnes*-Driven Disease Pathology: Current Knowledge and Future Directions

**DOI:** 10.3389/fcimb.2017.00081

**Published:** 2017-03-14

**Authors:** Joerg R. Leheste, Kathryn E. Ruvolo, Joanna E. Chrostowski, Kristin Rivera, Christopher Husko, Alyssa Miceli, Martin K. Selig, Holger Brüggemann, German Torres

**Affiliations:** ^1^Department of Biomedical Sciences, NYIT College of Osteopathic MedicineOld Westbury, NY, USA; ^2^Molecular Pathology Division, Massachusetts General Hospital and Harvard Medical SchoolBoston, MA, USA; ^3^Department of Biomedicine, Aarhus UniversityAarhus, Denmark

**Keywords:** *Propionibacterium acnes*, sarcoidosis, BPH, prostate cancer, Parkinson disease

## Abstract

This review discusses the biology and behavior of *Propionibacterium acnes* (*P. acnes*), a dominant bacterium species of the skin biogeography thought to be associated with transmission, recurrence and severity of disease. More specifically, we discuss the ability of *P. acnes* to invade and persist in epithelial cells and circulating macrophages to subsequently induce bouts of sarcoidosis, low-grade inflammation and metastatic cell growth in the prostate gland. Finally, we discuss the possibility of *P. acnes* infiltrating the brain parenchyma to indirectly contribute to pathogenic processes in neurodegenerative disorders such as those observed in Parkinson's disease (PD).

## General overview

**Figure 1:**
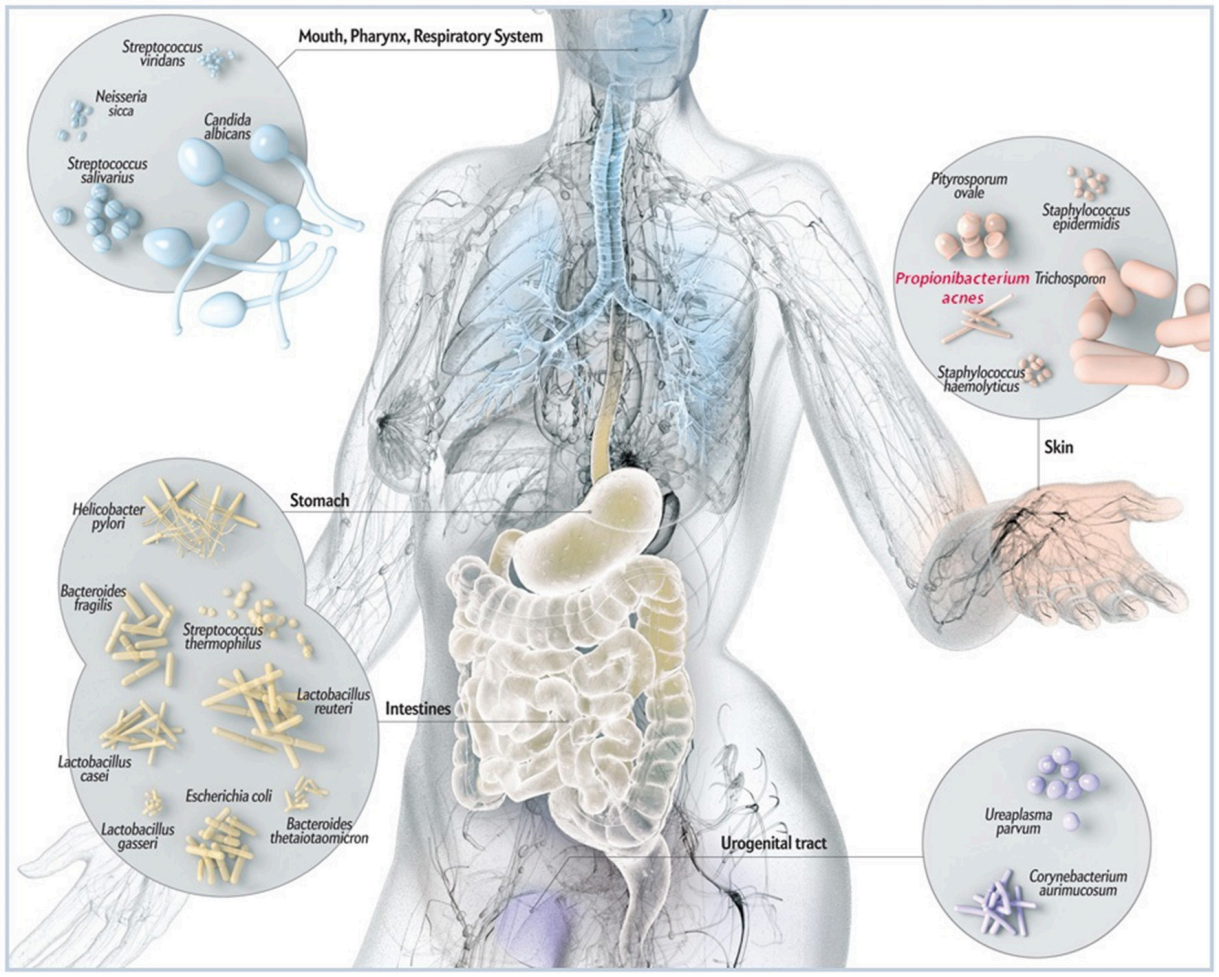
**Schematic illustration of common microbes found at barrier sites in humans including *P. acnes***. While *P. acnes* is present on all external and internal surfaces (i.e., oral and gastrointestinal epithelia, conjunctiva), it is most prevalent on the human skin. There it resides in hair follicles of the face and back where it is associated with the common skin disease *acne vulgaris*. By most, *P acnes* is still considered a mostly benign and commensal microorganism, however, reports about its malicious opportunistic side are increasing. Adapted and with kind permission from Bryan Christie Design (http://bryanchristiedesign.com/).

**Figure 2:**
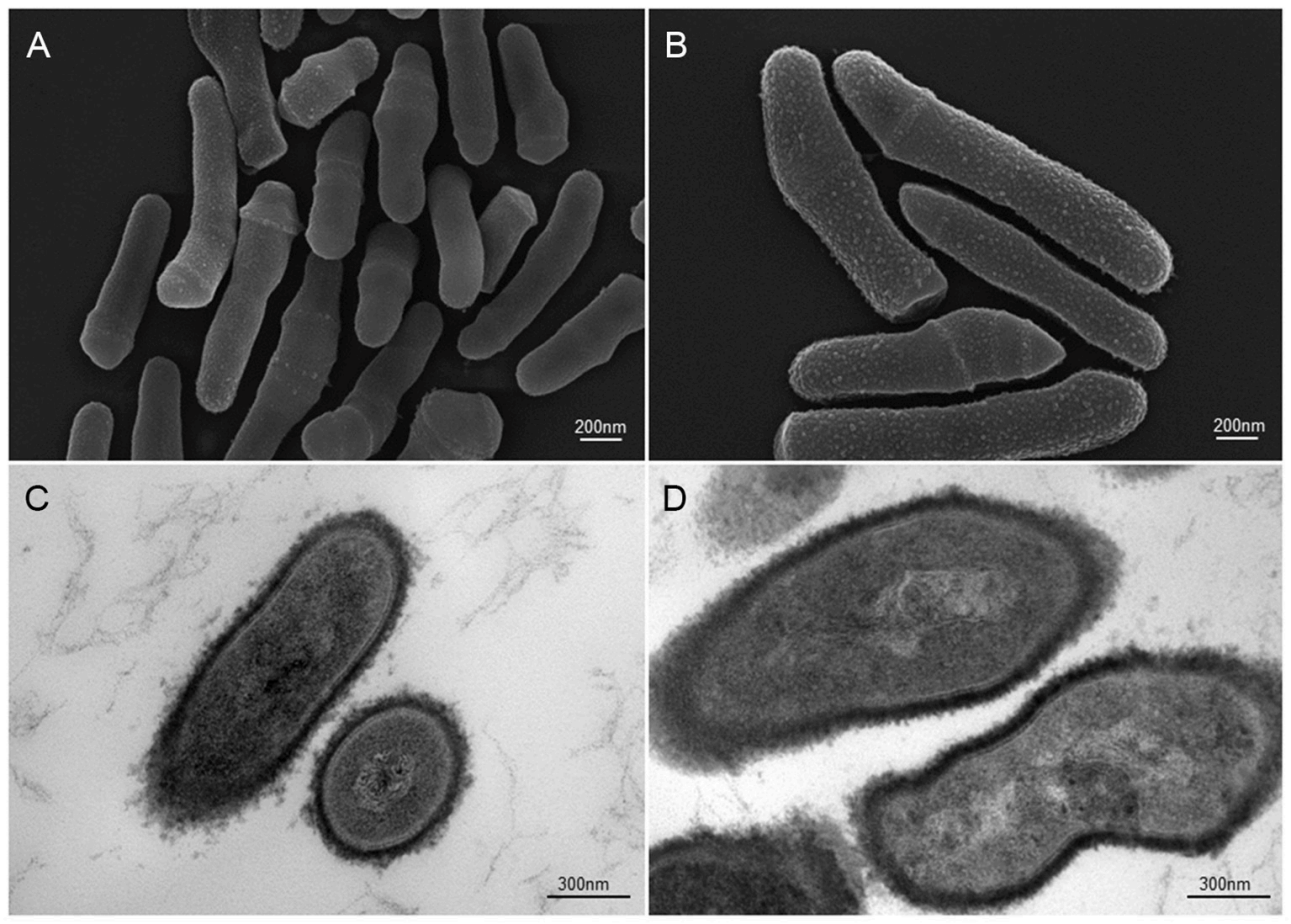
**Scanning and transmission electron microscopic (SEM and TEM) images of *P. acnes* strain KPA (A, B** = SEM; **C**, **D** = TEM). Recent advances in isolation and culturing techniques are revealing that *P. acnes* infections have been grossly underestimated shedding a new light on this opportunistic bacterial species. Microscopy by Volker Brinkmann, Max Planck Institute for Infection Biology, Berlin, Germany. Scale bar = 200/300nm.

A large fraction of microorganisms not only reside within us but also live on us. Indeed, the human skin harbors a heterogeneous mix of mostly non-pathogenic bacteria, fungi and viruses that probably contribute to skin surface health (Figure 1). *Propionibacterium acnes* (*P. acnes*) is a ubiquitous, slow growing, rod-shaped, non-spore forming, Gram-positive anaerobe (Figure 2) found across body sites, including sebaceous follicles of the face and neck (Funke et al., 1997; Grice and Segre, 2011; Findley and Grice, 2014). It is often considered part of our commensal microbiota at barrier sites (Cogen et al., 2008) which is established *via* mechanisms of adaptive immune tolerance during the early neonate period (Scharschmidt et al., 2015). Although the topographical distribution of the anaerobe in sebaceous sites is significant, the spatial and personal distribution of *P. acnes* is more individual-specific than site-specific (Oh et al., 2014). Moreover, the biogeography and individuality of *P. acnes* is highly dynamic as changes in health or changes in pH, temperature, moisture and/or sebum content may also affect the range of niches occupied by the microorganism (Grice et al., 2009). Similar to the distribution of skin microbes, skin conditions can also shape the function of *P. acnes* in terms of pathogen expansion in disease. For example, *P. acnes* has been linked to skin insults such as acne vulgaris in the face and neck, and progressive macular hypermelanosis on the back (Bojar and Holland, 2004; Kurokawa et al., 2009; Barnard et al., 2016). In addition, certain disease-associated phylotypes of *P. acnes* have the ability to persist on body implants and surgical devices causing a wide-range of post-operative infectious conditions, such as endocarditis, endophthalmitis and intravascular nervous system infections (Perry and Lambert, 2011; Portillo et al., 2013). The untoward features of *P. acnes* also extend to the prostate gland where tissue invasion and intracellular deposition of the bacterium has been frequently noted in glandular epithelial cells and circulating macrophages; a phenomenon thought to indirectly contribute to benign prostate hyperplasia (BPH) and prostate cancer (Tanabe et al., 2006; Alexeyev et al., 2007; Fassi-Fehri et al., 2011; Mak et al., 2012; Bae et al., 2014; Davidsson et al., 2016). However, it is not clear what the underlying mechanisms used by *P. acnes* are to induce infection, inflammation and/or metastasis outside the skin. What is known with some certainty is that bacteria-infected keratinocytes, sebocytes and/or adipocytes secrete several pro-inflammatory chemokines and cytokines as well as anti-microbial factors (e.g., cathelicidin) hinting at specific disease mechanisms (Graham et al., 2004; Nagy et al., 2006; Lee et al., 2010; Zhou et al., 2015; Sanford et al., 2016; Yu et al., 2016).

## Sarcoidosis

Sarcoidosis is a disease of unknown etiology that leads to inflammation in organs as diverse as lungs, liver, skin and lymphatics. A putative link of sarcoidosis with *P. acnes* was first proposed when the bacterium was isolated from sarcoid lesions of the skin and lymph nodes (Eishi et al., 2002; Yamada et al., 2002). These findings have been significantly corroborated (de Brouwer et al., 2015), and further expanded by various *in vitro* experiments demonstrating the invasion capacity of *P. acnes* in HEK293T (human embryonic kidney) and A549 (human alveolar epithelial carcinoma) cell lines (Tanabe et al., 2006). Most recent work in sarcoid broncho-alveolar lavage (BAL) fluid and cells showed significant upregulation of a *P. acnes*-specific immune response (Schupp et al., 2015). In addition, experiments in mice have shown that viable *P. acnes* can induce pulmonary granulomas similar to those observed in sarcoidosis patients (Werner et al., 2017). A general overview detailing the link between sarcoidosis and *P. acnes* is further given by Eishi (2013).

Studies attempting to characterize the signaling pathways activated by *P. acnes* during infection showed that nuclear factor-kappaB [NF-κB], a transcriptional factor that regulates the expression of genes involved in immune and inflammatory cascades is activated by *P. acnes* (Kim et al., 2002). More broadly, toll-like receptor 2 (TLR2) was shown to be a critical receptor for the NF-κB-dependent response to *P. acnes*, revealing the capacity of this bacterium to provoke the selective activation of innate immunity genes (Inohara and Nuñez, 2001; Chamaillard et al., 2003; Moreira and Zamboni, 2012). Furthermore, the role of host genetics was examined in sarcoidosis cases that were associated with *P. acnes* infection. Single nucleotide polymorphisms (SNPs) in the nucleotide-binding oligomerization domain (NOD) of proteins NOD1 and NOD2 were correlated with *P. acnes* infection among 73 sarcoidosis patients with 52 interstitial pneumonia and 215 healthy controls (Tanabe et al., 2006). NOD1 and NOD2 are intracellular pattern recognition receptors that can sense bacterial molecules such as peptidoglycan moieties. Along the same lines, *in vitro* experiments have shown that internalization of *P. acnes* into HEK293T cells can result in activation of NOD1 and NOD2, suggesting a disease mechanism based on chronic inflammation or local immunosuppression (Tanabe et al., 2006). These findings also suggest that invasive *P. acnes* can act as bacterial ligands to cause aberrant NOD receptor activation in certain individuals with long-lasting susceptibility to sarcoidosis. However, future experiments have to clarify the exact mechanistic chronology of whether or how *P. acnes*-mediated aberrant NF-κB activation may induce granuloma formation in a NOD1/NOD2-dependent manner.

Although invasive *P. acnes* could be a possible etiology of sarcoidosis and perhaps other diseases, elucidating causation and correlation between *P. acnes* infection and pathology is murky as bacterial strain heterogeneity, host genetics as well as host's environments must be considered whenever a study links a microbiome to a disease state.

## Benign prostate hyperplasia (BPH) and prostate cancer

Chronic or recurrent inflammatory processes have long been implicated in the progression of BPH and prostate cancer (De Marzo et al., 1999; Nelson et al., 2004; Sfanos et al., 2014). Inflammation is attributed to the presence of specific biomarkers such as elevated interleukin (IL)-6, tumor necrosis factor alpha (TNFα) and the acute phase protein, C reactive protein (Mechergui et al., 2009; Menschikowski et al., 2013; Yu et al., 2015). Recent work on urological fluids (i.e., urine, seminal fluid, prostatic secretions) as well as prostate biopsies suggests a significant conditional shift toward certain microbial species which may be used as a diagnostic index (Yu et al., 2015; Ni et al., 2016).

**Figure 3:**
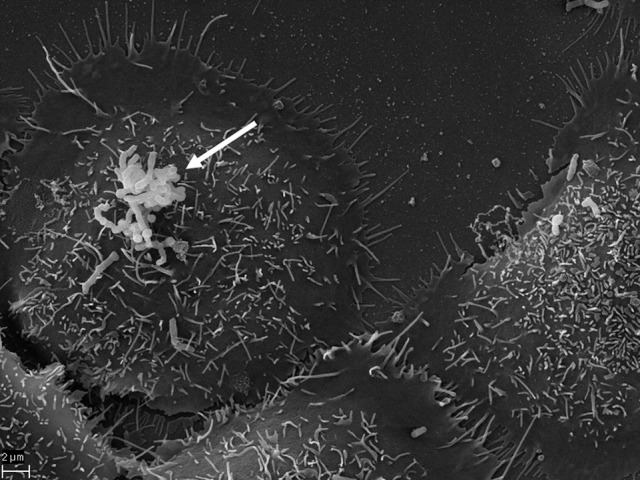
**SEM of *P. acnes* strain P6 (arrow) *in vitro* on cultured prostate epithelial cells RWPE1**. The prostate epithelial cell-invasive behavior of *P. acnes* is well documented in both, *in vivo* and cell-based studies where a vimentin-mediated invasion process looks likely (Mak et al., 2012). Microscopy by Volker Brinkmann, Max Planck Institute for Infection Biology, Berlin, Germany. Scale bar = 2 μm.

A good amount of work is suggesting that the specific association of *P. acnes* with the prostate and the invasion of prostate epithelial cells in particular (Figure 3) may contribute to the pathology of BPH or prostate cancer with an inflammatory component (Sfanos et al., 2013; Davidsson et al., 2016). However, it is currently unclear whether *P. acnes* represents a true infectious agent of the prostate, a commensal or accidental prostate microbion. It is plausible that prostate-located *P. acnes* are derived from the skin that are accidentally introduced, for instance during a prostate biopsy—a viewpoint that should raise concerns with certain diagnostic workup scenarios.

**Figure 4:**
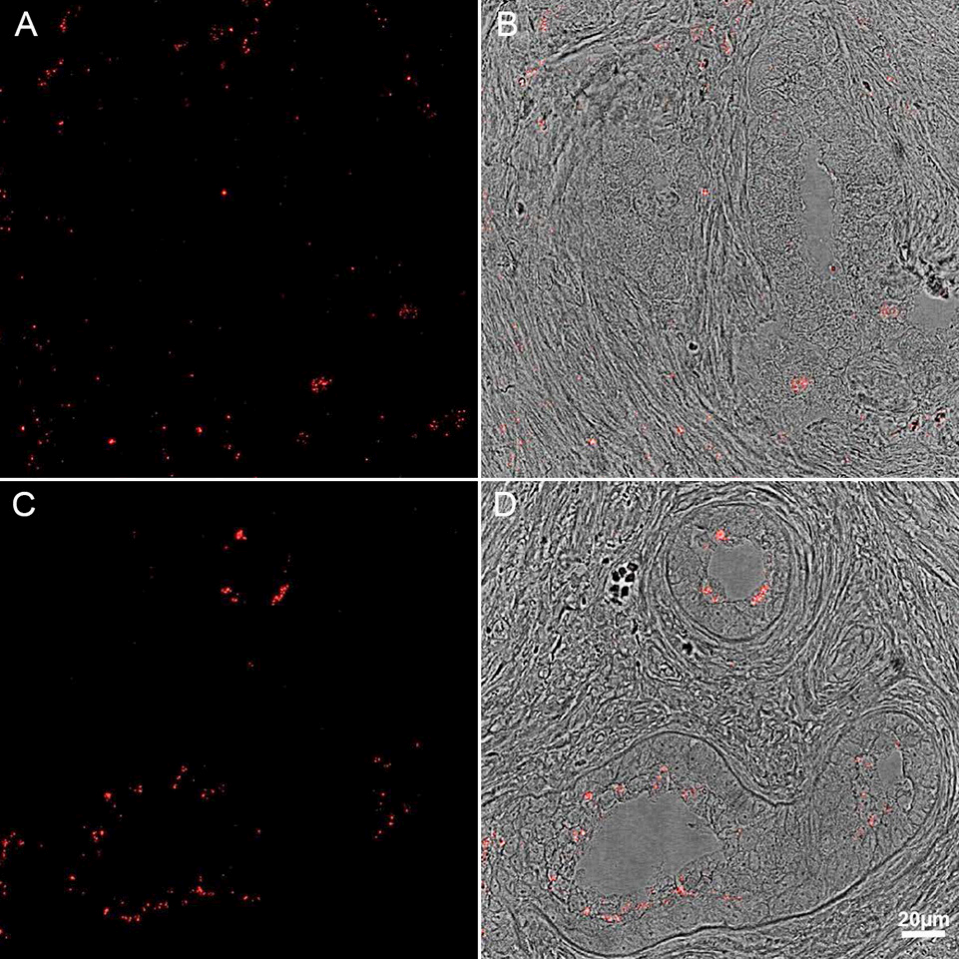
**Immunohistochemistry of human prostate tissue samples stained with *P. acnes* antibody (red)**. Adapted with permission from Fassi-Fehri et al., 2011 (Supplementary Figure 2B). Presence of *P. acnes* in human prostate tissue samples with benign prostatic hyperplasia **(A,B)**; or adenocarcinoma **(C,D)**. Extensive bacterial load was detected in both cases.

Whatever the route of entry or pathogenic potential, a significant number of prostate tissues obtained through transurethral resection for BPH, or radical prostatectomy for cancer, were previously tested positive for *P. acnes* aggregates apparently residing within roving macrophages (Alexeyev et al., 2007; Bae et al., 2014). Additional reports based on human samples have provided further evidence for a link between BPH or prostate cancer and *P. acnes* using various technical approaches, including cultivation, confocal microscopy for visualization of the bacterium and *in situ* hybridization, immunohistochemistry (Figure 4) and PCR-based profiling of bacterial 16S rRNA (Hochreiter et al., 2000; Cohen et al., 2005; Sfanos et al., 2008; Fassi-Fehri et al., 2011; Bae et al., 2014; Davidsson et al., 2016). Further evidence comes from animal studies indicating that inoculation of *P. acnes* into the murine or rat prostate and bladder leads to an overt, long-term inflammatory response and a wide-range of cellular disturbances within the prostate gland (Olsson et al., 2012; Shinohara et al., 2013).

Attempts to phylogenetically analyze disease-associated *P. acnes* strains from cancerous prostate glands have revealed that most prostate isolates belong to phylogenetic clades that are rare on human skin, indicating that a skin-derived contamination during sampling is unlikely (Mak et al., 2013; Davidsson et al., 2016). Along the same lines, similar inflammatory pathways as those described for sarcoidosis, including NF-κB, IL-6, STAT3 and COX2, appear to be activated by *P. acnes* both under *in situ* and *in vitro* conditions (Drott et al., 2010; Fassi-Fehri et al., 2011; Mak et al., 2013; Tsai et al., 2013; Bae et al., 2014). Although the precise etiology for these inflammatory changes is not yet clear, several membrane-bound pattern recognition receptor pathways are broadly distributed in mammalian urinary and genital systems that avidly recognize bacterial and viral components (Jorgensen and Seed, 2012; Gambara et al., 2013). These host cell receptors, for example TLRs, promote cytokine production which is a core feature of innate immunity against microbial pathogens. Collectively, these findings suggest that through their capacity to trigger various aspects of immunity, invading *P. acnes* can act as primary driver and/or amplifier of disease severity.

## Spondylodiscitis and back pain

One of several post-operative complications involving *P. acnes* is inflammation of the intervertebral disk and the surrounding intervertebral space (diskitis) following discectomy (Harris et al., 2005). Concomitant degenerative infection of adjacent vertebrae (spondylodiscitis) can be a common feature and the root cause for serious neurological damage and pain if treatment is delayed (Uçkay et al., 2010). Aside from unintentional surgical introduction right into the vertebral column, pathogens can also arrive through the arterial and venous spinal blood supply (hematogenous spreading). While *Staphylococcus aureus, Escherichia coli*, and *Proteus species* are most commonly isolated, *P. acnes* is the most abundant anaerobic pathogen in this context and likely underreported due to culturing challenges. There has been some clinical evidence that patients with herniated nuclear (nucleus pulposus) disk material infected with anaerobic pathogens in general, and *P. acnes* in particular, are more likely develop inflammation and edema of the adjacent vertebrae (Modic changes type I) and back pain (Albert et al., 2013; Urquhart et al., 2015). Clinical and animal-based follow-ups are now corroborating the initial findings showing that local *P. acnes* proliferation causes upregulation of inflammatory markers and disk degeneration consistent with Modic changes (Aghazadeh et al., 2016; Dudli et al., 2016). There is now even first clinical evidence to suggest that bacterial infection of the intervertebral disk with *P. acnes* and/or *Staphylococcus epidermidis* may actually precede all other issues as the root cause of disk herniation and associated pathological changes (Rajasekaran et al., 2017).

## Parkinson's disease (PD)

**Figure 5:**
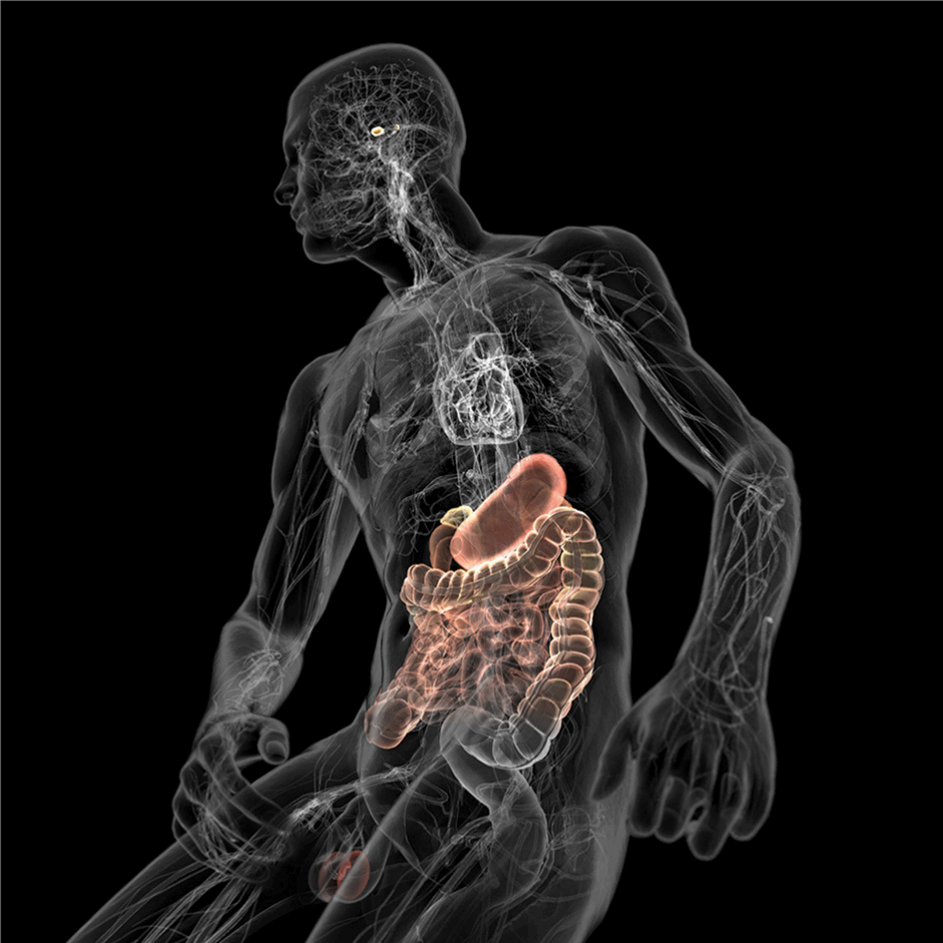
**Schematic illustration of the gut-brain axis (colored) with the microbe-filled digestive system on one end and the brain with its homeostasis centers (hypothalamus and pituitary) on the other**. Both are connected through the cardiovascular and autonomic nervous system including the vagus nerve (cranial nerve X) which could facilitate microbial transition into the central nervous system (CNS). Kindly with permission from Bryan Christie Design (http://bryanchristiedesign.com/).

The degenerating brain is characterized by neural system damage that may be attributed to atypical aggregation and deposition of mutant or misfolded proteins (e.g., Lewy bodies and Lewy neurites) as clearly documented in idiopathic PD (Taylor et al., 2002). What is generally not appreciated is that the brain is also susceptible to invading pathogens ranging from viruses and bacteria to fungi. These pathogens, or more specifically their endogenous components and/or metabolites, can produce central neurological deficits ranging from subtle signs of dementia and dystonia, which result from chronic, recurrent infection (De Chiara et al., 2012; Bibi et al., 2014), to more severe motor neuron disease as for example observed with the human endogenous retrovirus K (Li et al., 2015). Thus, it is clear that humans have a tremendously heavy systemic burden of microbes (Potgieter et al., 2015; Spadoni et al., 2015) which may incidentally contribute to the pathology of progressive neurodegenerative diseases with atypical protein component. Certainly, this working hypothesis is gaining considerable support as cognitive, emotional or pathological behavior appear to be indirectly affected by the spatial and personal distribution of microbes acting through the gut-brain axis (Figure 5) (Collins et al., 2012; Dinan et al., 2013; Mayer et al., 2014; Burokas et al., 2015). Indeed, a recent case-control study demonstrated that microbial variation in the gastrointestinal tract, both between and within individuals; correspond most significantly to phenotypical variations in PD (Scheperjans et al., 2015; Vizcarra et al., 2015). Although the underlying mechanisms linking microbiota composition with differences in PD are not clear, the above finding might at least explain the high prevalence of gastrointestinal abnormalities seen in PD patients (Dobbs et al., 2016). Further linking the microbiota to PD severity, sigmoid mucosal biopsies and fecal material collected from PD patients showed the presence of opportunistic and pro-inflammatory bacterial species that often cause chronic constipation, irritable bowel syndrome and ulcerative colitis (Keshavarzian et al., 2015). Collectively, these initial results suggest that relationships between different microbial communities in the gut are a common comorbidity in PD, and that unique individual signatures of the gut ecosystem can reinforce classic motor impairments of PD.

**Figure 6:**
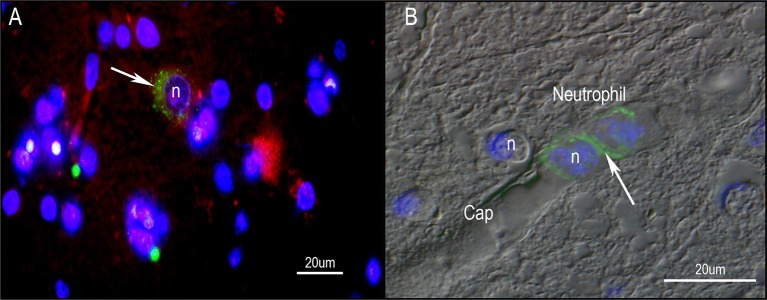
**Immunohistochemistry of human PD midbrain tissue samples stained for *P. acnes* (green; A+B), neuronal microtubules (MAP2; red; A) and nuclei (DAPI; blue; A+B)**. Age-linked lipofuscin auto-fluorescence was extinguished with Sudan Black B. Presence of *P. acnes* (arrow) in the periplasmic space of a human neuron **(A)** between nucleus (n) and cytoskeleton; or neutrophil **(B)** with its characteristic multi-lobed nucleus inside a midbrain capillary (Cap). These findings are typical for PD and absent in most control sections. Retrograde movements along cranial nerves, trauma-induced micro-bleeds as well as the newly discovered glymphatic system represent potential microbial pathways into the CNS.

Against this background, the question to be asked is whether there is any evidence that *P. acnes* plays any role in the pathophysiology of PD. For this possibility to occur, two conditions must be met: (1) *P. acnes* infection must precede the occurrence of classic symptoms of the disease such as shaking, motor initiation and slowness of movement; and (2) *P. acnes* inoculation must sufficiently induce symptoms of PD and/or lead to the loss of dopamine projection neurons in the midbrain nucleus known as *substantia nigra pars compacta*. So far, our laboratory has detected clusters of *P. acnes* localized to neurons of the midbrain and nearby cortical structures of autopsied PD brains (Figure 6). This unexpected finding adds further credence of *P. acnes* indirectly contributing to local bouts of inflammation similar to those seen in sarcoidosis and BPH. An open question is the extent to which *P. acnes* can indirectly enhance certain pathological features of PD. As PD is a highly heterogeneous disorder, it is unlikely that one disease mechanism applies to all PD phenotypes. Nevertheless, it is intriguing to speculate that resident skin microbes could initiate or amplify PD progression through inflammatory and/or genetic predisposition factors.

If *P. acnes* can gain access to dopamine cells in the midbrain, what is the most likely route of inoculation and infection? Although the skin is physically compartmentalized from the brain, cross-inoculation remains a risk factor. For example, the nares could potentially harbor pathogenic *P. acnes* strains which would then translocate to the brain parenchyma to hyper-activate local macrophages called microglia; a phenomenon that is a consistent feature of PD pathology (reviewed by Chao et al., 2014). Research into nosocomial infections demonstrate bacterial species as diverse as *Pneumococcus* sp. (Zwijnenburg et al., 2001) and *Salmonella* sp. (Bollen et al., 2008) roving readily along the olfactory nerve (cranial nerve 1; CN 1) and eventually into the olfactory bulb. Another potential route of entrance for harmful microbes or dangerous misfolded proteins is the vagus nerve (cranial nerve 10; CN 10). Indeed under some circumstances, misfolded aggregates in the form of Lewy bodies and Lewy neurites appear to migrate from the vagus nerve to the brain, providing further evidence for pathogenic bacteria in association with toxic proteins to potentially contribute to selective neuronal vulnerability (Holmqvist et al., 2014). Of interest, brain nuclei of CN 1 and CN 10 are among the first sites to preferentially show deposition of Lewy bodies and Lewy neurites during the course of PD progression (Braak et al., 2004). This particular observation not only highlights a likely route of entrance for bacteria into the brain, but also provides a potential mechanism for the observation that truncal vagotomy significantly decreases the PD risk (Svensson et al., 2015). The link between head trauma and PD (Harris et al., 2013; Jafari et al., 2013; Pearce et al., 2015), together with the notion of a diverse blood microbiome (Potgieter et al., 2015), implies a vast potential for pathogenic opportunism as traumatic breaches in the blood brain barrier occur. With the recent discovery of a brain lymphatic (glymphatic) system (Iliff et al., 2012; Hitscherich et al., 2016) another potential route of brain infection must be considered.

Taken together, there is preclinical and some clinical evidence to indirectly implicate *P. acnes* as an independent variable affecting PD incidence or severity. This evidence is complemented by reports associating a severe skin disorder, *acne inversa*, with Alzheimer's disease (Wang et al., 2010). From a therapeutic perspective, the possibility of *P. acnes* driving or amplifying core features of PD represents an important resource for antibiotic approaches to neurodegenerative diseases. And from a research perspective, it would be worthy to examine patients with skin disorders more closely for evidence of neuropathology with earlier onset and a more severe disease phenotype.

## Future outlook

The review presented in this article outlines an unexpected dark side of *P. acnes* with respect to pathology. Intracellular persistence of *P. acnes* is implicated in diseases of the lungs and prostate gland and possibly the brain. This is a clear testimony for the pathogenicity of skin-derived bacteria in certain disease phenotypes. Further investigation will need to focus on several looming questions in *P. acnes* biology: (1) what are the crucial *P. acnes* interactions in disease susceptibility? (2) What are the mechanisms used by *P. acnes* to influence disease progression? (3) If certain populations of *P. acnes* can enhance susceptibility to disease severity, are there other configurations of *P. acnes* that are protective? (4) Proof of concept that *P. acnes* infection directly associates with disease pathology, or more specifically, does the study of concept show causation or just correlation? And finally (5) Can *P. acnes*-driven disease pathology be treated with conventional antibiotic therapy and diagnostic applications?

## Author contributions

JL, KER, JC, KR, CH, AM, HB, and GT prepared the manuscript. JL and MS prepared the figures.

## Funding

Intramural funding to JL was provided by NYIT College of Osteopathic Medicine.

### Conflict of interest statement

The authors declare that the research was conducted in the absence of any commercial or financial relationships that could be construed as a potential conflict of interest.
